# A commentary of “Cryo-EM achieves atomic resolution” in 10 remarkable discoveries from 2020 in *Nature*

**DOI:** 10.1016/j.fmre.2022.01.014

**Published:** 2022-01-31

**Authors:** Hong-Wei Wang

**Affiliations:** Ministry of Education Key Laboratory of Protein Science, Tsinghua-Peking Joint Center for Life Sciences, Beijing Advanced Innovation Center for Structural Biology, School of Life Sciences, Tsinghua University, Beijing 100084, China

## Abstract

A founding principle of structural biology is that, once researchers can directly observe macromolecules at sufficient resolution, it should be possible to understand how their 3D structures confer their biological functions. In two studies published concurrently in the October 2020 issue of the journal *Nature*, Yip et al. [1] and Nakane et al*.*[2] reported the sharpest images yet obtained by using single-particle cryo-EM, enabling the location of individual atoms in a protein to be determined for the first time. Both teams used improved hardware that broke the previous resolution limitations of cryo-EM imaging. With the development of these technologies, the increased signal-to-noise ratio of cryo-EM images will expand the technique's applicability. Combining these technologies may make it possible to determine biological structures with cryo-EM at a resolution even beyond 1 Å (0.1 nm) — an achievement that was near impossible in the past.①

The famous physicist Richard Feynman proposed in his famous 1959 lecture “There's Plenty Room at the Bottom” that “to make more rapid progress in biology is to make the electron microscope 100 times better.” More fundamental questions of biology could be answered by being able to see finer biological structures. At that time, the maximum resolution of electron microscopes was 1 nm, which was not enough to distinguish individual atoms in the microscopic world. It was even more challenging to use electron microscopes to distinguish atoms in biological structures.

Structural biology developed very rapidly over the half-century after Feynman's lecture. Scientists have been able to analyze biological macromolecule structures at atomic resolution using X-ray crystallography and nuclear magnetic resonance spectroscopy rather than electron microscopy. X-ray crystallography uses the principle of X-ray diffraction through highly ordered three-dimensional crystals of biological macromolecules to determine their structures. A key requirement is to obtain highly ordered three-dimensional crystals. Nuclear magnetic resonance spectroscopy measures the changes in the specific nuclear spin state of biological macromolecules in response to high-energy magnetic fields to analyze the structure of molecules in solution. It requires a relatively high concentration of the sample and a relatively long time for data collection and is primarily suitable for analyzing biological macromolecules with relatively small molecular weight. Electron microscopy uses direct magnification imaging of microscopic objects to observe their structures, which has the advantages of small sample requirements and suitability for analyzing various structural states. However, the application of electron microscopy for high-resolution to determine the structure of biological samples faces several major technical hurdles, including how to maintain water-containing biological samples in a high-vacuum electron microscope, how to maintain the structure of biomolecules effectively under high-energy electron radiation, and how to effectively extract weak signals of biological structures in electron microscope imaging.

After years of continuous efforts, cryo-electron microscopy has been established since the 1970s. In this technology, water-containing biological samples are rapidly cooled to the temperature of liquid nitrogen and embedded in vitreous ice, thereby fixing the structure of biological macromolecules in the liquid state immediately before freezing. Biological samples that were frozen in liquid nitrogen maintain their hydrated state in the electron microscope's high vacuum and are more resistant to electron radiation damage, so electron microscopy can be used to observe their structures. Since the invention of the electron microscope, its imaging capability has been greatly improved after decades of continuous improvement, breaking through an imaging resolution of 1 Å at the beginning of this century and allowing analysis of the structure of inorganic materials at the atomic level. This has benefited from innovation and improvement in several electron microscopy optical elements and computer control software, such as the field-emission electron gun, multi-level condensers, stable objective lens systems, spherical aberration (Cs)-corrected devices, and energy filters. However, when analyzing the structure of frozen biological samples at high resolution using electron microscopy, it is also necessary to solve the technical problem of extracting the structural signal of biological macromolecules. Imaging of frozen biological samples using electron microscopy requires a much lower dose of electron radiation than inorganic materials, resulting in a very low signal-to-noise ratio in the image. This has been the main problem preventing cryo-EM from playing a major role in the field of structural biology.

In the past two decades, two technological innovations have greatly improved the resolution of cryo-EM structural analysis, making it the most powerful research technique in structural biology. The first is the invention of the direct electron detector, which allows the microscope to respond directly to electrons to record a digitized image, thereby improving the high-efficiency transmission of the image signal. This device can also acquire high-speed, multi-frame images in the same sample field, eliminating sample drift-induced signal loss through digital image processing and improving the image quality. Multi-frame image acquisition allows the electron irradiation intensity of the sample to be analyzed, which is particularly important for the observation of frozen biological samples by electron microscopy. The second technological innovation is the invention of a new cryo-EM image processing algorithm. Statistics-based image processing algorithms developed over the past few decades play an important role in improving the signal-to-noise ratio of cryo-EM images of biological macromolecules and have gradually developed into single-particle cryo-EM methods. At the beginning of this century, the concept of probability statistics was introduced into the field of single-particle cryo-EM. The concept was quickly found to be well suited for solving the problem of low signal-to-noise ratio in cryo-EM images, and quickly applied in many aspects of cryo-EM image processing. These two technological innovations coincided at the right time and complemented each other, pushing the resolution of cryo-EM from 8–10 Å to 3–4 Å in just 2–3 years and realizing the “resolution revolution” of cryo-EM. Since the publication of the high-resolution structure of TRPV1 in 2013, the number of near-atomic resolution structures resolved by single-particle cryo-EM has increased exponentially, and the resolution has also been improved over the years. More importantly, cryo-EM could resolve the structures of many complicated biological macromolecular complexes that could not be resolved using X-ray crystallography or nuclear magnetic resonance spectroscopy. The essential mechanisms of several major biological processes have been unveiled by cryo-EM. Cryo-EM is only one step away from allowing direct observation of the atoms in biological macromolecules.

These two studies in 2020 opened the door to the era of atomic-resolution cryo-EM. Similar to the previous breakthroughs, the two studies are still benefiting from innovations in electron microscope technology. The two works proved that improved electron guns, spherical aberration (Cs)-corrected devices, energy filter imaging systems, and image processing software and algorithms could effectively improve the resolution of biological macromolecular structures determined by cryo-EM. At a resolution of 1.2 Å, single-particle cryo-EM can not only clearly resolve the spatial positions of the carbon, oxygen, and nitrogen atoms in each amino acid of the protein molecule, but also the spatial coordinates of hydrogen atoms. Although hydrogen atoms can already be observed at sub-angstrom resolution using crystal diffraction technology, this is the first time that hydrogen atoms of biological molecules have been observed in an amorphous state using single-particle cryo-EM, which is of more biochemical significance. In the past, scientists have always believed, based on experience from crystallography, that the resolution of single-particle cryo-EM must exceed a resolution of 1 Å to achieve the goal of resolving hydrogen atoms. Now the experiments have proved that the goal can be achieved by cryo-EM at a resolution of 1.2 Å. Feynman's dream from over 60 years ago is finally realized. The molecular structures closest to the biological environment can be finely revealed and analyzed using atomic-resolution cryo-EM and then correlated with their functions to understand these biological macromolecules’ structural changes and regulatory mechanisms. Based on this, structural biology research can be conducted more extensively, the patterns in biological phenomena can be revealed more deeply, and new drug molecules can be developed more effectively.

## Declaration of Competing Interest

The author declares that he has no conflicts of interest in this work.
